# Spatiotemporal Distribution of PRRSV-1 Clades in Hungary with a Focus on the Era of Disease Eradication

**DOI:** 10.3390/ani14010175

**Published:** 2024-01-04

**Authors:** Ádám Bálint, Szilvia Jakab, Eszter Kaszab, Szilvia Marton, Krisztián Bányai, Sándor Kecskeméti, István Szabó

**Affiliations:** 1 Veterinary Diagnostic Directorate, National Food Chain Safety Office, H-1143 Budapest, Hungary; kecskemetis@nebih.gov.hu; 2 National Laboratory for Infectious Animal Diseases, Antimicrobial Resistance, Veterinary Public Health and Food Chain Safety, H-1143 Budapest, Hungary; jakab.szilvia@vmri.hun-ren.hu (S.J.); kaszab.eszter@vmri.hun-ren.hu (E.K.); marton.szilvia@vmri.hun-ren.hu (S.M.); 3 HUN-REN Veterinary Medicinal Research Institute, H-1143 Budapest, Hungary; 4 One Health Institute, Faculty of Health Sciences, University of Debrecen, H-4032 Debrecen, Hungary; 5 Department of Pharmacology and Toxicology, University of Veterinary Medicine, H-1078 Budapest, Hungary; 6 National PRRS Eradication Committee, H-1024 Budapest, Hungary; iszabodr@t-online.hu

**Keywords:** porcine reproductive and respiratory syndrome, disease eradication, phylogeny, genetic diversity, molecular epidemiology, PRRS

## Abstract

**Simple Summary:**

Porcine reproductive and respiratory syndrome virus (PRRSV) can be controlled with vaccines and biosecurity measures. Uncovering local strain diversity is expected to help better understand the disease epidemiology and viral evolutionary dynamics, which will contribute to more effective disease control and prevention. In Hungary, although sporadic virus genetic information has been available since the 1990s, a more systematic data collection was initiated only in the mid-2000s and was maximized after the eradication program started in the mid-2010s. In this study, we report on the origin and spread of co-circulating genetic lineages and clades of PRRSV in Hungary till 2020. This longitudinal molecular epidemiological survey was an important element of the national disease eradication program to achieve PRRS-free status in our country.

**Abstract:**

Porcine reproductive and respiratory syndrome (PRRS) is the cause of the most severe economic losses in the pig industry worldwide. PRRSV is extremely diverse in Europe, which poses a significant challenge to disease control within a country or any region. With the combination of phylogenetic reconstruction and network analysis, we aimed to uncover the major routes of the dispersal of PRRSV clades within Hungary. In brief, by analyzing >2600 ORF5 sequences, we identified at least 12 clades (including 6 clades within lineage 1 and 3 clades within lineage 3) common in parts of Western Europe (including Denmark, Germany and the Netherlands) and identified 2 novel clades (designated X1 and X2). Of interest, some genetic clades unique to other central European countries, such as the Czech Republic and Poland, were not identified. The pattern of PRRSV clade distribution is consistent with the route of the pig trade among countries, showing that most of the identified clades were introduced from Western Europe when fatteners were transported to Hungary. As a result of rigorous implementation of the national eradication program, the swine population was declared officially free from PRRSV. This map of viral diversity and clade distribution will serve as valuable baseline information for the maintenance of PRRSV-free status in the post-eradication era.

## 1. Introduction

Porcine reproductive and respiratory syndrome (PRRS) is a major disease of pigs that causes severe economic losses worldwide [1,2]. The disease is caused by PRRS virus (PRRSV), an enveloped, single-stranded RNA virus of the genus *Betaarterivirus*, family *Arteriviridae* [3]. The PRRSV genome is approximately 15 kilobases in length and contains 11 partially overlapping open reading frames (ORFs). ORFs 1a and 1b comprise 80% of the genome and encode non-structural proteins responsible for transcription, replication and immunomodulation. Eight ORFs (2a, 2b, 3–7, and 5a) encode the viral structural proteins [4]. Based on their 30–45% genetic distance, PRRSV strains can be classified into two distinct species, *Betaarterivirus suid 1* (PRRSV-1) and *Betaarterivirus suid 2* (PRRSV-2) [3,5].

Clinical signs of the disease range from reduced reproductive performance in sows to acute respiratory diseases in young piglets. The immunosuppressive effect of PRRSV in combination with other pathogens within the herd significantly increases morbidity and mortality [6]. Specific treatments for PRRS are not available. Control of PRRS can be achieved by limiting the adverse effects of the disease at various stages of production using vaccines and biosecurity measures, but the most effective method of elimination of PRRSV in infected herds is complete eradication. In the United States, the American Association of Swine Veterinarians takes the lead in PRRSV eradication [7]. In Europe, four countries are free from PRRSV (Norway, Sweden, Finland and Switzerland), and local eradication programs have been launched in Denmark and the Netherlands [8,9]. In Scotland, eradication was extended to the national level in 2018 [10]. In Hungary, due to the prevalence of PRRSV infection and the associated economic losses, a national PRRSV eradication program was launched in 2014 based on territorial principles which was approved by the EU’s competent veterinary committee. The program resulted in Hungary’s pig population becoming officially PRRSV-free by the end of 2022 [11,12,13].

PRRSV is extremely diverse in Europe, which makes the control of PRRS a significant challenge even within a single country or region [14]. The genetic classification is based on a rather short, variable genomic region, ORF5. The basis of classification is the clustering of sequences in phylogenetic trees. Three main PRRSV-1 lineages (lineage 1, 2 and 3) and multiple clades within lineages (mainly within lineages 1 and 3; lineage 1a to 1g and lineage 3a to 3g) have been reported to occur in Europe [14]. The geographic distribution of lineages and clades was summarized by Balka and co-workers by collecting data available up to 2014. This report has shown, for example, that lineage 1 occurs throughout Europe, whereas clades 1c, 1d and 1e have been reported only in East European countries. Clade 1g has occurred mainly in Spain and Germany. Lineage 2 has circulated chiefly in Denmark, but it has been also reported from East Europe and outside Europe. Two lineage 3 clades, 3e and 3g, have been described in Italy, and another clade, 3f, has been identified in some central European countries [14].

The launch of national PRRS eradication program in Hungary was the starting point for a large-scale molecular epidemiological investigation based on ORF5 sequencing of circulating strains. The present study reports the occurrence and evolution of PRRSV genetic clades in Hungary, the spatial distribution of their occurrence and the traceability of infection in individual pig units. Data have been accumulated since the mid-1990s, but here, we focus mainly on the era of disease eradication.

## 2. Materials and Methods

### 2.1. History of PRRS Epizootiology in Hungary

In Hungary, the history of the PRRS can be divided into three periods with significantly different characteristics.

- 1996–2004. In this period, Hungarian pig farming was characterized by the nearly equal (i.e., 50–50%) distribution of fattening pig production between backyard and large farms, the very strict hierarchical animal health administration system and the import of live animals limited to a small number of breeding animals. In this period, PRRSV was probably introduced through breeding animals, but the laboratory infrastructure and diagnostic tests to diagnose the disease were in their infancy. Additionally, sequencing of the ORF5 genomic region did not help to determine the route of infection.
- 2004–2014. Hungary became a member of the European Union. As a consequence for the pig industry, the import of live pigs increased dramatically (to nearly 90,000 tons in 2 years). In 2011, more than one million live pigs were imported to Hungary. Simultaneously, there was a significant decrease in the number and production of small-scale herds. The constant reorganization of the animal health administration, relegation of epidemic prevention and softening of animal transport and quarantine rules have favored the spread of PRRS. In 2008, PRRS became a notifiable disease again in Hungary.
- 2014–2022. This period was defined by elimination efforts and preservation of the results achieved. The 3/2014 (I.16.) VM decree of Minister of Rural Development has settled the legal background for the National PRRS Eradication Program coordinated by the National PRRS Eradication Committee, a unique proceeding in the EU at the time. According to the National PRRS Eradication Plan, that regulated the tasks, all pig farmers had to certify their herd based on serological (ELISA) and virological (PCR) diagnostic tests for PRRSV carried out by the National Reference Laboratory at the Veterinary Diagnostic Directorate, National Food Chain Safety Office. Besides the elimination of PRRS from Hungarian pig herds, another principal goal of the eradication plan was to identify the origin of PRRS infections for all PCR-positive herds by epidemiological investigation. The main laboratory tool of this effort was the sequence analysis of the ORF5 gene, as described elsewhere in detail [15,16,17]. In this period, very rigorous field epidemiology investigations were carried out to trace the origin and potential intra-country transmission of vaccine strains and to identify imported strains as well as farm-specific resident strains. It is clearly not possible to share all details in this study; therefore, further data are available upon request.

### 2.2. Classification by Phylogenetic and Network Analysis

Our routine molecular epidemiological investigation was performed using ORF5 sequences from all three periods. Detailed amplification and sequencing protocols can be found in previous reports [16,17]. However, for the first and second periods, retrospective analyses with limited conclusions could only be made because we did not have information on the circumstances under which different virus strains were introduced into pig farms or because the pig farm in question had ceased to exist over time. Consequently, the most comprehensive surveillance data are available from the third period (2014–2022), which spanned nearly a decade. 

At the end of 2020, a total of 2900 ORF5 sequences were available in our local sequence database. Of these, 261 sequences were downloaded from GenBank. This dataset included relevant reference sequences generated from field strains and vaccine strains (including Porcilis, MSD AH.; Amervac, Hypra; Unistrain, Hypra; Reprocyc, Boehringer Ingelheim; Suvaxyn, Zoetis). The remainder (*n* = 2639) were collected from samples from large-scale breeding farms (*n* = 470 farms), large-scale fattening farms (*n* = 307 farms) and some backyard farms in Hungary. All sequences were derived from diagnostic specimens (including but not limited to blood, various organ samples, fetuses, and lymph nodes). Importantly, many sequences (*n* = 832) were found to be of vaccine origin. All samples used to generate sequence data were collected as part of routine monitoring and from outbreaks where suspected PRRS cases were reported. 

In this study, only a subset of representative sequences was included in a way to best reflect all PRRSV variants from every pig holding settlement between 2003 and 2020. This sample set contained 301 full-length or nearly full-length ORF5 sequences. In each herd, the index sequence was kept in the analysis. The index sequence was considered the first sequence within each evolutionary variant in the same herd. If a new PRRSV emerged in a farm from external source that did not cluster within the previous farm-specific sequences, and showed more than 2% difference at nucleotide level (https://www.pig333.com/articles/use-and-interpretation-of-sequencing-in-prrsv-control-programs_9091 (accessed on 10 December 2023)), this sequence was also included in the phylogenetic studies as a new index sequence. Along with the Hungarian ORF5 sequences, other reference PRRSV-1 strains (*n* = 99) were also included from international reports, which helped in the demarcation of genetic clades.

Multiple sequence alignments were generated in AliView software [18]. Pairwise nucleotide identities were calculated in Mega X [19]. Genetic relationships among representative sequences of each unique submission were established by similarity network analysis and phylogenetic analysis. Similarity networks were created as described earlier [20]. The phylogenetic tree was generated on the IQ-TREE web server (http://www.iqtree.org/ (accessed on 10 May 2023)), implementing the best-fit model GTR+F+R5 with branch support analysis of 1000 ultrafast bootstrap iterations. With the phylogenetic and network analyses, we aimed to demonstrate the spread of PRRSV clades (or virus strains) of the same origin within Hungary by clustering PRRSV ORF5 sequences. Therefore, we defined a clade as a group of different PRRSV sequence variants that can be traced back to a common ancestor on their lineage. Classification of the ORF5 sequences into Hungarian clades was in accordance with the previously determined PRRSV-1 lineages and clades [14]. 

## 3. Results

### 3.1. Old and New PRRSV-1 Clades in Hungary

**Figure 1:**
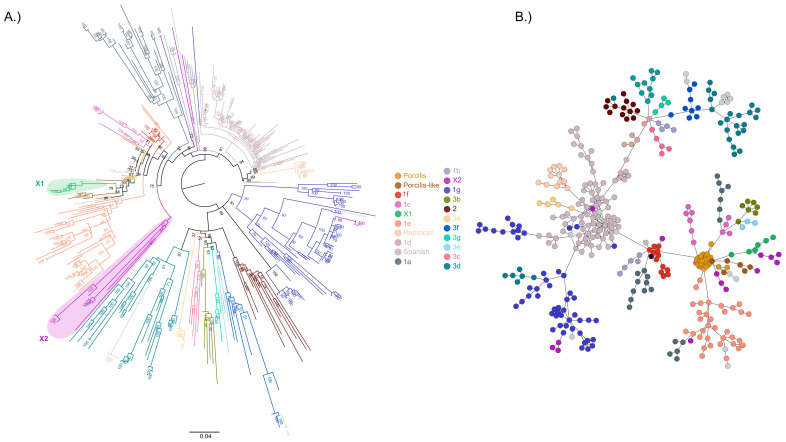
Phylogenetic analysis (**A**) and network representation (**B**) based on European reference (*n* = 99) and Hungarian ORF5 index sequences (*n* = 301) of PRRSV-1 (total number of sequences, 400). The different clades are marked with distinct colors, and the two novel clades, X1 and X2, are highlighted on the phylogenetic tree.

Between 2003 and 2020, over 2600 ORF5 sequences were determined as part of routine PRRSV-1 strain surveillance. The majority of sequences (80%) were generated after the launch of the national eradication program. Published studies from Europe identified 3 lineages and 14 (1a–1g, 3a–3g) clades within lineage 1 and 3 of PRRSV-1. The majority of these clades were also detected in Hungary. Moreover, two novel clades were also identified (Figure 1). 

We restricted the analysis to the 301 study sequences that were considered representative concerning study area (and pig holdings) and calendar year. These clades included clade 1a to 1g, 2, 3c, 3d, and 3f. Other old unassigned clades were named as follows: Porcilis, Porcilis-like, Spanish, and Reprocyc. Furthermore, two novel clades, X1 and X2, were also designated. The clades genetically most closely related to these new clades were 1f and Porcilis, with mean inter-clade genetic distances ranging from 7% to 13% and 6.6% to 21.6%, respectively, with clades X1 and X2. Index sequences (or haplotypes) within clades were most numerous within clade ‘Spanish’ (*n* = 69) and clade 1g (*n* = 56). The least heterogenous clades were clade 3c and 2, with only two major sequence variants. The most diverse clades were X2 and 3d, while the most conserved were clade 1f and the vaccine strain origin clades (Table 1). The members of clades 1d and 3a are primarily disseminated in Poland and Romania, respectively, while clades 3b, 3e and 3g are found in Italy [14]. These PRRSV clades were not identified in our country, most likely due to the lack of significant pig trade from these countries to Hungary. 

### 3.2. Spatiotemporal Distribution of PRRSV-1 Clades

**Figure 2:**
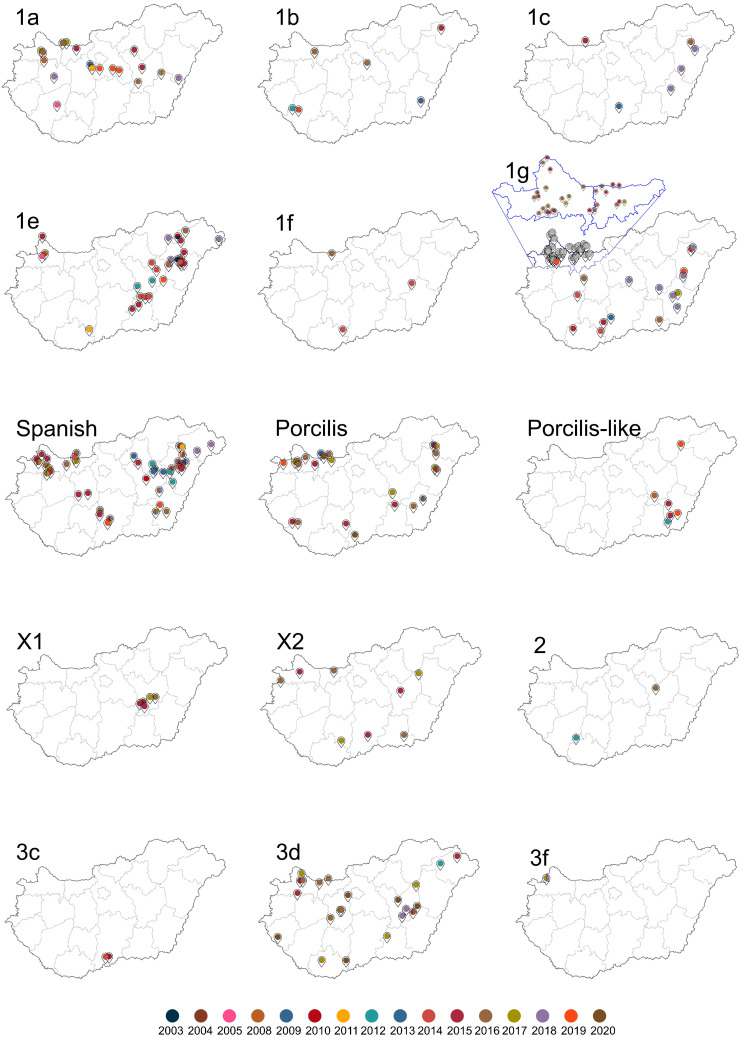
Spatiotemporal distribution of PRRSV-1 lineages and clades between 2003 and 2020, Hungary. The labels above each map indicate the lineage and clade designations.

Altogether, the presence of 16 clades was confirmed by phylogenetic or network analysis along with epidemiological investigation applied in this country-wide investigation (Figure 2). This section reports the events characterizing and shaping the distribution of the specified clades which occurred in Hungary from 2003 to 2020. We focused on the period 2014–2020, when the national eradication program was extended in Hungary.

#### 3.2.1. Clade 1a

PRRSV-1 strains belonging to this clade first appeared in 2005 in the Transdanubia region (settlements affected: Kaposvár, Pápa, Adony, and Besnyő) (representative strains: HU08/2005, HU41/2008, 30640/2011, and 3712/2013); however, their origin could not be tracked in this early surveillance period. In 2015 and 2017, clade 1a PRRSVs were introduced from two sources, one into Komárom-Esztergom county and another into Győr-Moson-Sopron county with imported fattening stocks from the Netherlands (the anonymized locations were “S” and “E” for the two distinct Dutch sources, respectively) (Figure 2). The virus originated from the Dutch “S” farm in 2015 and remained in the receiving farm (24102/2016); however, on one occasion it was introduced to another fattening farm with illegal animal transport (39942/2018). In 2017, from the same source in the Netherland, the virus was re-introduced (15841/2015) through unknown transport routes to a farm in Békés county (8588/2018, 3922/2018, 62397/2017, 4779/2017, 6968/2017), where additional PRRSV strains were in circulation simultaneously. The virus imported from the Netherlands to pig farm “E” in 2017 (27477/2017) appeared later at a fattening pig unit, at a distant location in Pest county (2019), through illegal animal transport (26940/2019, 31094/1/2019, 25177/2019). Afterwards, a breeding unit that had been free of PRRS for many years became infected during transport through a slaughterhouse. A transport vehicle of the same slaughterhouse was very likely the source of PRRS infection in a herd of native Hungarian pigs, in the same year.

#### 3.2.2. Clade 1b

Regarding clade 1b (Figure 2), the first source of PRRSV was probably the previously infected boars imported from Western Europe to a PRRSV-free sow herd in 2010. This virus spread when the index herd shared animals to the fattening unit of a multi-site swine farm (Farm “A”) in 2012 (3340/2012). Unfortunately, the sequence of the founder virus strain imported from abroad was not available for comparison. Subsequently, two distinct variants of clade 1b PRRSV were detected in the breeding unit of the same multi-site swine farm in 2014 (40551/2014, 29919/2014). These two ORF5 sequences showed 4.1 and 4.3% nucleotide difference, respectively, to the PRRSV detected in 2012. In 2016, a new variant appeared (68195-20/2016), again at the same farm, showing 1.6% difference when compared to the earlier strain, 29919/2014.

In 2015, there was a PRRSV outbreak in a farrow-to-finish type pig farm (Farm “B”), located 487 km far from Farm “A”. Sequence analysis of the 3671/2015 collected from Farm “B” showed high nucleotide similarity (96%) to 40551/2014 from Farm “A”. Among the two pig farms, there was no swine, swine-origin or feed transport, and, for this reason epidemiological investigation was conducted in order to find out the possible route of transmission. As revealed by field epidemiology investigation, Farm “B” was visited several times in the pre-infection period by Farm “A” management; hence, the source of infection presumably was related to human activity, but the exact way remained unknown. Moreover, ORF5 sequences detected in the proximity of Farm “B” did not share high identity with 3671/2015, a fact that further confirmed our assumption.

In 2016, clade 1b PRRSV was detected in a large-scale fattening farm (Farm “C”) located 215 km north of Farm “A”, when a stock that arrived from farm “A” was tested. The sequence (63817/2016) of this farm was identical to the sequence (68195/2016) from Farm “A”.

In 2016, another clade 1b PRRSV outbreak occurred in a distinct multi-site swine farm (Farm “D”), where the identified sequence (represented by 30860/2016) showed 98.1% nucleotide identity with 29919/2014 from Farm “A”. The distance between the two farms was 265 km, and epidemiological investigation uncovered the involvement of an inadequately disinfected transport vehicle of a local slaughterhouse that operated for these farms.

#### 3.2.3. Clade 1c

The exact origin of the first detected PRRSV (6324/2009) in clade 1c is not known; however, strains belonging to this clade infected several large-scale breeding units a decade later (9824/2018, 11045/2018) in Békés county. Additionally, with prefattener movement, other fattening units were infected in Békés (14908/2018) and Hajdú-Bihar (25733/2018) counties (Figure 2). 

In 2015, an independent introduction of clade 1c PRRSVs was identified (26485/2015) in a German import stock. Fortunately, strains from these infected pigs did not spread to any other farms.

#### 3.2.4. Clade 1e

PRRSV-1 strains classified into clade 1e could be divided into two distinct genetic groups within Hungary. The majority of clade 1e PRRSVs were linked to farms in Hajdú-Bihar county (Figure 2); this group was represented by HU01/2003 and HU02/2003, two early strains from 2003. In addition, mutually but independently, other clade 1e PRRSVs were detected in Jász-Nagykun-Szolnok county in 2012 (660/2012).

The PRRSV-1 strain HU01/2003 emerged to infect a farrow-to-nursery type farming unit (8442/2012) in the close proximity but a decade later; this virus subsequently spread to many different pig-producing farms (54741/2014, 34851/2015, 22925/2015, 37410/2015, 41925/2015, 48427/2016). The main causes of this large-scale dissemination of the PRRSV in the affected area were the following: neglecting external disease control measures, inappropriate sanitation of the operating vehicles, and topographical proximity of the affected pig farms.

The strain HU02/2003, which was closely related to the HU01/2003, became widely spread in Hungary. It emerged in Győr-Moson-Sopron county (e.g., HU17/2005) and Csongrád county (12543/2014, 38461/2014, 50500/2014, 65575/2015) by the trade of fattening pigs and construction crews simultaneously working on previously PRRSV-free sites. For the same reason, further pig farms were infected in 2018 (52901/2018, 38417/2018).

A clade 1e PRRSV (64113/2015) detected in a large breeding herd in Hajdú-Bihar county that evolved from an early strain (HU31/2004) had a long journey. It was transferred by the personnel to a herd, located in Győr-Moson-Sopron county, that had been free of the virus for a longer period of time (65824-1/2015).

The other group of clade 1e PRRSVs spread mainly in Jász-Nagykun-Szolnok county and the bordering Csongrád county, mainly through fattening units (660/2012, 9470/2012, 46360/2014, 48580/2014, 1304/2015).

#### 3.2.5. Clade 1f

The prototype strain of PRRSV-1, Lelystad, belongs to the clade 1f. In Hungary, a total of three outbreaks were linked to this clade (23917/2014, 29128/2014, 22632/2016) (Figure 2), and all of these were found in imported fattening pigs which originated from the Netherlands.

#### 3.2.6. Clade 1g

PRRSVs belonging to clade 1g were derived from three main sources, the Netherlands, Germany and Slovakia.

Clade 1g PRRSV-1, similar to clade 1a, was introduced and emerged in Hungary due to the import of Dutch prefattening pigs. This scenario applied to several strains detected in 2014–2016 (30838/2014, 54292/2014, 6325/2015, 7083/2015, 20519/2015, 65131/2015, 10621/2016, 30838/2016, 32089/2016, 66238/2016). Another clade 1g PRRSV (6101/2017) was introduced with weaned piglets imported from the Netherlands in 2017. With the infected stock, the virus spread to a large-scale fattening farm and a breeding unit located in Békés county (28370/2017). Transport vehicles of a company responsible for the collection and transport of carcasses and additional transport of live animals aided the dispersal of this PRRSV into pig holdings in Veszprém county (13050/2/2019), Békés county (53965/2018, 53975/2018, 58480/2018) and Hajdú-Bihar county (5571/2018, 7104/2018, 3993/5/2019), likewise into Bács-Kiskun county (848/2018). 

A clade 1g PRRSV strain (5314/2013) probably arrived from Germany with breeding animals, and the personnel mediated its further dispersal.

A third line of import for clade 1g PRRSV-1 (20424/2013) was detected in a farrow-to-nursery type farm in Ács and was closely related to Slovakian-origin strain from 2007 (KC522643, 13M). Prefatteners were sold widely by this establishment, and as a consequence of pig trading within the country, this genetic variant of clade 1g PRRSV was widely distributed in Hungary between 2015 and 2017 (33266/2015, 42547/2015, 44158/2015, 44817/2015, 46678/2015, 49324/2015, 56523/2015, 4399/2016, 5229/2016, 5588/2016, 13653/2016, 19946/2016, 23084/2016, 24682/2016, 34474/2016, 35018/2016, 35020/2016, 35957/2016, 51570/2016, 12623/2017) (Figure 2).

The source of strains 50708/2014 and 55909/2015 was a Slovakian pig farming company, situated relatively close to the border. 

In 2018, a clade 1g PRRSV (10083-1/2018) of unknown origin infected two other pig farms in Békés county (20114/2018, 28320/2018) (Figure 2).

#### 3.2.7. Clade ‘Spanish’

PRRSVs of the Spanish clade were first reported from Hungary in late 1990s and arrived with pigs imported from Spain (e.g., U40690, KF666907, KF666936, KF666930). The affected pig farms in Hajdú-Bihar county (Figure 2) started intensive immunization against the disease with the then available Amervac MLV vaccine. This vaccine is based on strain VP-046 BIS, an important member of this clade. Afterwards, the Amervac PRRSV strain spread either when herds were vaccinated (18803/2011) or when rearing the purchased vaccinated stocks (55020/2015). These strains have evolved gradually, and these vaccine-derived field strains spread to several previously PRRS-free pig herds over a decade (HU09/2005, 5890/2012, 14701/2013, 41006/2014, 50908/2014, 28168/2018).

Interestingly, one representative of the Spanish clade (1293/2019) was introduced by fattening pigs which arrived from a Danish collecting station.

#### 3.2.8. Porcilis and Porcilis-like Strains

Porcilis sequences were routinely identified in farms where it was used as vaccine. In fact, at least 26.4% of ORF5 sequences we generated were of Porcilis vaccine origin [16].

Concerning the Porcilis-like sequences, the origin of the Porcilis-like clade is misleading based on genetic information alone, as the sequences were closely related to the Porcilis PRRS vaccine strain. Epidemiological data revealed that this clade emerged independently of the Porcilis clade and was introduced by imported breeding stocks, first detected in 2008 (9701/2008). Later, it spread to other breeding units within Hungary (9880/2012, 18002/2015). Inadequately implemented rules in live animal movement, in staff’s work and in personnel turnover, led to the transfer of this PRRSV to other pig holding facilities (57870/2015, 3865/2019, 25436/2019).

#### 3.2.9. Clade X1

The PRRSVs in this clade were all detected from different farms but with very close connections (identical owner, shared feed mixer, fattening stock placement from common source, etc.), situated in the same region. The transmission chain of the clade X1 started in 9908/2015; this event was followed by infection of two fattening herds and a breeding herd of another owner (23127/2015, 11617/2017, 23142/2015). The strain identified from the fattening farm in 2017 infected two other farms located in close proximity to each other that usually form an epidemiological unit (20482/2016, 38736/2016) (Figure 2).

#### 3.2.10. Clade X2

Clade X2 PRRSV-1 strains arrived in our country from the Netherlands and Germany with consignments of prefatteners. A PRRSV-infected Dutch import arrived at a fattening unit (1787/2017) and subsequently spread further to a farrow-to-finish type farm, which is separated only by a fence. Two other PRRSVs (1783/2017, 4266/2017) were detected in prefattener stocks which came from the same Dutch trader. Viruses (44844/2015, 7704/2016, 43171/2016) originating from Germany were also imported from a particular farm via the import of fattening material (Figure 2).

#### 3.2.11. Clade 2

One of the two PRRSV index sequences belonging to clade 2 was detected in an import shipment of breeding sows assigned to re-populate a completely renovated, disinfected pig farm of several hundred sows. The flock arrived from Denmark in 2012 and the Danish national veterinary acknowledged the Danish origin of the infection (12780/2012). The other index sequence, also imported with Danish fattening stock, belonged to a strain that had previously circulated in Hungary (54182/2016) (Figure 2).

#### 3.2.12. Clade 3c

The majority of clade 3c was disseminated in Serbia, a country bordering Hungary, and its emergence in Hungary resulted from significant local traffic (Figure 2). A breeding farm located close to the border was infected most probably in 2014, but we obtained the first PRRSV sequence from this farm only in 2015 (28593/2015). The epidemiological investigation revealed that the virus spread subsequently to another farm, as they shared a meat processing plant (PRRSV-1-1/2014).

#### 3.2.13. Clade 3d

The viruses of this clade arrived from the Netherlands and Germany in 2011 and 2012 with live animal shipments to Hungary (2090/2011, 16729/2012) (Figure 2).

Several PRRSVs were identified due to the activity of a company that continuously imported piglets from three Dutch farms for pre-rearing and sold them for further rearing in Hungary (28932/2016, 30443/2016, 28355/2016, 2105/2017, 53181/2016, 51533/2016, 53265/2016).

Two pig farming units were infected with members of this clade directly from Germany (27714/2017, 16546/2015), and the virus spread to a third pig farm (37665/2018) with a vehicle rented for managing the transport to a slaughterhouse.

Another PRRSV (36750/2017) classified in this clade originated from Denmark. A Hungarian pig-holding company purchased PRRS-free fattening pigs; however, the shipment arrived in Hungary indirectly, via a collection station, where the minimum conditions to prevent the spread of the virus were not in place, and as a result, the otherwise virus-free stock could have acquired PRRSV infection at this station.

#### 3.2.14. Clade 3f

Clade 3f includes PRRSVs of Czech and Slovak origin. Clade 3f PRRSV-1 strains (7624/2017, 47989/2018, 27779/2018) emerged in Hungary close to the Slovak border (Figure 2), in a spot where three owners’ pig farms are practically in the same plot. Upon field investigation, one owner reported that six years before the 2018 incident, an animal of unknown origin with PRRS infection was settled, and therefore it seemed to be reasonable to assume that this virus was in circulation in all three pig holdings.

## 4. Discussion

Despite the availability of distinct vaccines, challenges in the control of PRRS remained. One crucial pillar of disease control is the genetic characterization of circulating virus strains in order to identify the origin of infection and determine the routes of transmission between pig farms, within a geographical region or an administrative area. Sequencing one of the most variable genomic regions, the ORF5, is still a gold-standard procedure for surveying these issues [21,22,23,24,25,26,27,28,29,30,31,32,33,34,35,36,37,38,39,40]. The information obtained by means of molecular epidemiology further assists the improvement of external disease control measures of a given pig herd and provides the basis for reliable production of animal goods.

Classifying PRRSV into lineages or clades by phylogenetic analysis of the ORF5 supports the understanding of genetic relatedness among different PRRSV strains. However, classical representation by the bifurcating phylogenetic tree often fails to explain the exact genetic relationships if the genetic diversity of a large number of sequences is high [41]. To overcome this limitation and to facilitate PRRS control, it is worth considering building a minimum spanning phylogenetic network in addition to reconstructing a phylogenetic tree and then to adapt the results of field epidemiological investigations [42,43]. 

In a previous study [20], we published an improved minimum spanning phylogenetic network based on Hungarian PRRSV sequences collected during the eradication program and proved its suitability to conduct epidemiological investigations. In this study, we performed an extensive survey of circulating PRRSVs in Hungary, over a period of 17 years (2003–2020), in order to define their phylogenetic as well as epidemiological relationships, including the emergence and geographical spread of different genetic clusters. In 2021–2022, until freedom from wild-type PRRSV in Hungary, only sporadic introductions of PRRSV-1 strains were recorded, but all affected animals were culled in the quarantine; therefore, these strains were not included in this study. After gathering a representative ORF5 dataset, we classified the sequences into 3 lineages and 16 clades in accordance with previous reports [14]. Additionally, we defined two novel clades. Moreover, we analyzed the origin, the emergence as well as the spread of each clade during PRRS elimination. Although the National PRRS Eradication Program started legally in 2014, we also worked retrospectively with earlier sequences.

We confirmed our previous assumption that the most common source of PRRSV-1 in Hungary is infected pig stocks, predominantly prefatteners, coming from the European Union. Joining the EU opened the markets and permitted the free movement of goods, including animal products and live animals across countries. Hungary became a net import country of prefatteners, and the main suppliers for this were the Netherlands, Denmark and Germany. Similar findings were reported for PRRSV-2 in the same study period [44]. To reduce this tendency, the National Chief Veterinary has been regulating the importing practice, and from 2017, purchased pigs settled in for fattening must come from certified PRRS-free herds. This regulation has completely changed the direction of imports, as transaction with Dutch pig farmers has practically ceased. Unfortunately, the operation and use of collecting stations in Denmark remains a great threat for the Hungarian pig population. Another constitutive source of infection was found to be live animal movement within Hungary. In particular, the lack of disinfection of transport vehicles poses an increased risk of disease spread. Special attention should be paid to ensure the appropriate isolation of adjacent pig holdings and to treat them as a single unit for epidemiological management. Neglecting the personal sanitation during site visits could also contribute to the transmission of the virus among different establishments.

## 5. Conclusions

This study is based on the largest national collection of PRRSV sequences published so far. Over 2600 PRRSV-1 specific sequences were collected in the study period. Although the country has become officially PRRS-free, the available sequence data will serve as a useful database for future epidemiological preparedness during the maintenance period. We believe that the Hungarian PRRS eradication program would not have been possible, or would have been much less successful, if the results of the continuous, real-time evaluation and comparison of PRRSV ORF5 sequences diagnosed in the swine herds had not been available to the authorities and to the experts managing the program.

## Figures and Tables

**Table 1:** PRRSV-1 clades in Hungary and the genetic diversity among identified haplotypes.

Clade	First Detected (Year)	No. of Haplotypes	Nucleotide Identity Range within Clade (%)
1a	2005	21	86.3–99.7
1b	2012	6	84.5–96.4
1c	2009	9	89.3–100
1e	2003	37	87.8–99.8
1f	2014	3	99.5–99.8
1g	2013	56	82.2–100
Porcilis	2009	27	96–100
Porcilis-like	2008	6	92.2–98.8
Spanish	1990s	69	91.4–100
Reprocyc	2016	11	96.5–100
2	2012	2	89.9
3c	2014	2	90.9
3d	2011	28	80–100
3f	2017	5	92.7–100
X1	2015	6	96.2–98.5
X2	2012	13	80.9–100

## Data Availability

Sequence data have been deposited in GenBank; all new and existing records are as follows: OR828462–OR828525, MN102129–MN102159, MN102160–MN102200, MN102202–MN102208, MN102209–MN102243, MN102245–MN102280, MN102282–MN102315, MN102317–MN102327, MN102328–MN102334, MN150536, MN150537, MF600534, MT628991, MF600491, MF600497, MF600522, MN102244, MN102281, MN102316, MT628907, MT628934, MT628962, MT628992, MT628995, MT629000, MT629011, MT629014, MT629021, MT629022, MT629630. The same dataset is available as Supplementary File attached to this paper.

## Supplementary Materials

The following supporting information can be downloaded at: https://www.mdpi.com/article/10.3390/ani14010175/s1, Sequence File S1: ORF5_sequences.
